# Estimating EQ-5D and OAB-5D health state utilities for patients with overactive bladder

**DOI:** 10.1186/1477-7525-11-200

**Published:** 2013-11-19

**Authors:** Katia Desroziers, Samuel Aballéa, Khaled Maman, Jameel Nazir, Isaac Odeyemi, Zalmai Hakimi

**Affiliations:** 1 Creativ-Ceutical USA Inc, Chicago, IL, USA; 2 Creativ-Ceutical SARL, Paris, France; 3 Creativ-Ceutical Ltd, London, UK; 4 Astellas Pharma Europe Ltd, Chertsey, UK; 5 Astellas Pharma Global Development, Leiden, The Netherlands

**Keywords:** Health-related quality of life, Overactive bladder, EQ-5D, OAB-5D, Quality-adjusted life-years, Utility assessment

## Abstract

**Background:**

Limited utility data on patients suffering from overactive bladder (OAB) are available in the literature. The objectives of this study were to estimate utility values in patients with OAB using the generic EQ-5D questionnaire and the OAB-5D disease specific questionnaire, to investigate the relationship between utilities and symptoms, and to evaluate the sensitivity of the two instruments to changes in symptom severity.

**Methods:**

Analyses were based on pooled data from three large multicenter randomized 12-week placebo-controlled trials (SCORPIO, ARIES, CAPRICORN). Patients completed a micturition diary, EQ-5D and OAB-q (a quality of life questionnaire from which OAB-5D is derived) at baseline and at weeks 4, 8 and 12. Time trade-off tariffs elicited from UK population were applied to obtain utilities from both instruments. Repeated measures regressions were used to estimate EQ-5D and OAB-5D utilities by micturition frequency and incontinence severity level. As a test of sensitivity of the instruments, utility changes from baseline to week 12 were estimated by symptomatic response (improvement, stable or worsening).

**Results:**

The sample included 4427 patients. Mean utilities (± standard deviation) across all visits were 0.82 (±0.21) for EQ-5D and 0.86 (±0.09) for OAB-5D. Correlation between EQ-5D and OAB-5D was 0.34 (p < 0.0001). Both OAB-5D and EQ-5D utilities increased as OAB symptoms improved. Utility values were similar for severe levels of symptoms, but higher with OAB-5D than with EQ-5D for mild cases. Micturitions and incontinence had similar impact on EQ-5D utilities, but micturitions had greater impact on OAB-5D utilities than incontinence. Changes from baseline in OAB-5D utilities differed significantly according to symptomatic response. Changes in EQ-5D utilities were not significantly associated with changes in micturition frequency and weakly associated with changes in incontinence severity among patients with mild symptoms at baseline.

**Conclusions:**

This study showed that both EQ-5D and OAB-5D can detect changes in severity of OAB, especially in severe cases. However, OAB-5D is more sensitive than EQ-5D in measuring differences between treatments in milder cases. Both OAB-5D and EQ-5D–although leading to different results–may be useful to derive utilities from clinical trial data and perform cost-effectiveness analyses.

**Trial registration:**

Clinical Trials NCT00689104, NCT00662909, NCT00912964.

## Background

Overactive bladder (OAB) is a chronic condition defined by the International Continence Society as urinary urgency, with or without urgency incontinence, usually with increased daytime frequency and nocturia [1]. Urgency is a sudden, compelling and difficult to defer desire to pass urine. Symptoms can have significant impact on a person’s health related quality of life (HRQoL), interfering with daily routines, causing embarrassment as well as lowered self-esteem. OAB is also associated with an increased risk and prevalence of co morbidities, including falls and fractures, urinary tract and skin infections, and depression [2].

Previous reports from the European Prospective Investigation into Cancer and Nutrition (EPIC) study estimated the overall prevalence of OAB in Europe and Canada to be 12.8% among women and 10.8% among men [3].

Estimates of health utilities associated with different states of OAB are required for economic evaluations of OAB treatments. Utilities represent individual’s preferences for specific health states. They are measured on a scale where 0 represents death and 1 perfect health. According to the methodological guidelines of the UK National Institute for Health and Clinical Excellence (NICE) [4] and the US Panel on Cost-Effectiveness in Health and Medicine [5], quality adjusted life years (QALYs) should be obtained by assigning values elicited from the general public, to health states described by persons with OAB, by means of a generic classification of health states. NICE specifically advises the utilisation of EQ-5D, a widely used generic HRQoL instrument. Sets of utilities were generated for EQ-5D health states in many countries using choice-based methods, such as time trade-off (TTO) or standard gamble.

Only few data on utilities in OAB are available. EQ-5D TTO utilities were obtained in a Swedish willingness to pay survey among patients with urge incontinence [6,7]. This survey showed a correlation between EQ-5D utility and urinary symptoms, measured as the sum of the numbers of micturitions and leakages over one day. However, Yang et al. considered OAB as a condition for which the generic dimensions of EQ-5D might be irrelevant or insensitive for capturing small though important clinical changes [8]. EQ-5D has a number of potential limitations, such as the lack of sensitivity in specific disease contexts [9-11].

To overcome these limitations, Yang et al. developed a 5-dimensional preference-based disease-specific measure, named OAB-5D [see Additional file 1] and derived from the Overactive Bladder Questionnaire (OAB-q) [8,12]. The OAB-q is a validated and widely used questionnaire to assess symptom bother as well as HRQoL in OAB patients [13-15]. Even though OAB-q was not originally designed to measure utilities, Yang et al. built a model for deriving utilities from the 5 items forming the OAB-5D system, based on a survey in the UK general population, using the TTO method.

The objectives of this study were to estimate utility values in patients with OAB using the generic EQ-5D questionnaire and the OAB-5D disease specific questionnaire, to investigate the relationship between utilities and symptoms, and to evaluate the sensitivity to the changes in OAB symptoms of both preference-based instruments based on data from three pooled clinical trials.

## Methods

### Patients and study design

This post-hoc analysis was based on pooled data from three 12-week, randomized, double-blind, parallel group, placebo controlled, multi-centre studies (SCORPIO [16], ARIES [17] and CAPRICORN [18]) assessing the efficacy against placebo of mirabegron, a first in class beta 3 adrenoreceptor agonist, in the treatment of patients with symptoms of OAB. The three clinical trials had similar inclusion and exclusion criteria. Four thousand six hundred and twenty-two (4622) patients were randomized in the three trials conducted across Australia, Europe, United States of America and Canada. Male and female patients ≥18 years of age were screened for enrollment into the studies if they had OAB symptoms for at least 3 months. Patients were excluded if they had clinically relevant stress incontinence or mixed stress/urgency incontinence with stress as the predominant factor; an indwelling catheter; evidence of a symptomatic urinary tract infection, chronic inflammation, bladder stones, previous pelvic radiation therapy, previous or current malignant disease of the pelvic organs; or severe hypertension. At baseline, patients must have experienced an average of ≥8 micturitions/24 hours and ≥3 urgency episodes with/without incontinence over a 3-day period. Patients underwent a 2-week placebo run-in period followed at randomization by a 12-week phase where 1384 patients were administered placebo, 433 patients mirabegron 25 mg, 1379 patients mirabegron 50 mg, 931 patients mirabegron 100 mg, and 495 the anti-muscarinic tolterodine. A 3-day micturition diary, a generic HRQoL questionnaire (EQ-5D) and a disease-specific HRQoL questionnaire (OAB-q) were completed at baseline and weeks 4, 8 and 12. These analyses were based on pooled full analysis sets, which consisted of all randomized patients taking at least one treatment medication dose.

### Clinical measures and severity of symptoms

Patients completed a micturition diary over the 3 days preceding each study visit. From the diary, the following endpoints were derived: mean number of micturitions per 24 hours, mean number of urgency episodes (grade 3 and 4) per 24 hours, mean number of incontinence episodes per 24 hours and mean volume voided per micturition. For micturitions frequency and incontinence episodes, 5 severity levels were defined roughly based on the distribution (quintiles) of event observed in the pooled trials, using all visits combined. For the severity incontinence, class 1 (less severe) was composed of continent patients (no incontinence episode reported in the 3-day micturition diary).

### Preference based instruments

The EQ-5D instrument consists of 5 questions, relating to mobility, self-care, usual activities, pain/discomfort and anxiety/depression, with 3 possible answers for each item, corresponding to “no problem”, “moderate problems” or “extreme problems”. It can be used to calculate a utility index score on a scale on which 0 represents a health state equivalent to death and 1 represents full health. The UK TTO tariff was used to derive EQ-5D utilities in this study [19].

The OAB-q is a 33-item questionnaire consisting of an 8-item Symptom Bother scale and a 25-item HRQoL scale that has four subscales: coping, concern, sleep, and social interaction [13]. Responses are based on a six-point Likert scale. The OAB-q has demonstrated good internal consistency, reliability, test–retest reliability, concurrent validity, discriminate validity, and responsiveness to treatment-related change [13-15].

To value OAB health states, five items were chosen from the OAB-q to construct a new health state classification system (OAB-5D) namely: urge to urinate, urine loss, sleep impact, coping strategy, and concern with OAB, where each dimension had five levels of severity with level 1 denoting no problem and level 5 indicating an extreme problem [8]. A total of 3125 health states can be defined by the OAB-5D classification system (compared to 243 with EQ-5D), and all OAB-q data which contain these five items can be mapped to a specific OAB-5D health state.

### Statistical analysis

Utility scores derived from EQ-5D and OAB-5D were first described as means and standard deviations and then estimated by symptom severity. The Spearman correlation coefficient between EQ-5D and OAB-5D utilities was estimated.

For each type of symptom–micturition frequency and incontinence episodes–a linear regression model was used to estimate EQ-5D and OAB-5D utilities by symptom level, adjusting for gender, age and study as fixed effects, and geographical region as random effect. The correlation between utility values for one individual at different assessment visits was taken into account by means of a random patient effect. Using a similar method, differences between OAB-5D and EQ-5D utilities were estimated by symptom level, and tested for the null hypothesis of equal mean OAB-5D and EQ-5D utilities.

In addition, repeated measures linear models were used to predict mean OAB-5D and EQ-5D utilities according to severity levels of the 2 symptoms, with study identifier and several clinical and socio-demographic covariables. The listed covariates were removed from the models if not significant at 5%: gender, age, race, body mass index (BMI), type OAB (urgency incontinence, frequency or mixed), prior OAB surgery, previous OAB drug and duration of OAB symptoms.

Tobit and beta regression models were also estimated with repeated measurements random effect and the same covariates as above mentionned. Minimizing the Root Mean Squared Error (RMSE) was used as criterion to define the best model.

The purpose of this model was to provide a way to derive utilities from the micturition diary data, which are usually collected in trials of OAB treatments.

Finally, as a test of sensitivity of the instruments, utility changes from baseline to week 12 were estimated according to response in symptoms, defined as 3 possible items: “improvement higher than 1 level of symptoms”, “stable” or “worsening higher than 1 level of symptoms”. Linear models with covariables gender, age, study and response as fixed effects, and geographical region as random effect were estimated to provide adjusted means (SD) of utility changes from baseline to week 12 by response level. Models were run for overall population, patients with mild symptoms at baseline (level 1 to 3 in micturitions frequency for response in micturitions frequency, or level 1 to 3 in incontinence episodes for response in incontinence) and patients with severe symptoms at baseline (level 4 or 5 in micturitions frequency, or in incontinence episodes).

## Results

### Patient characteristics in pooled clinical trials

Across the three studies, the total number of patients was 4427, of which 3179 were female (71.8%). The majority of patients were from Europe (SCORPIO and CAPRICORN trials) with 1392 from Western Europe and 1095 from Eastern Europe (31.4% and 24.7% respectively). The remainders of the patients were from USA and Canada (ARIES and CAPRICORN trials) (39.0% and 4.8% respectively). Patients’ mean age was 59.4 (±12.9) years, 52.0% were previously treated with antimuscarinics. The baseline mean daily numbers of micturitions were 11.6 (±2.9), 11.7 (±3.4), 11.6 (±3.1) and 11.6 (±3.1) respectively for SCORPIO, ARIES, CAPRICORN and pooled trials (Table 1). The baseline mean daily numbers of incontinence episodes were 1.7 (±2.4), 2.1 (±2.7), 2.3 (±2.3) and 1.8 (±2.5) respectively for SCORPIO, ARIES, CAPRICORN and pooled trials (Table 1).

**Table 1:** Baseline demographic and clinical characteristics

**Patient characteristics**^**a**^	**N (%) or mean (±SD)**^**b**^			
	**SCORPIO (n = 1906)**	**ARIES (n = 1270)**	**CAPRICORN (n = 1251)**	**Pooled trials (n = 4427)**
Age	59.1 (±12.4)	60.2 (±13.4)	59.1 (±13.0)	59.4 (±12.9)
Female	1372 (72.0%)	950 (74.8%)	857 (68.5%)	3179 (71.8%)
BMI	27.8 (±4.9)	30.2 (±7.0)	29.4 (±6.4)	28.9 (±6.1)
Geographical region				
Western Europe	1906 (54.2%)	0 (0.0%)	359 (28.7%)	1392 (31.4%)
Eastern Europe	873 (45.8%)	0 (0.0%)	222 (17.8%)	1095 (24.7%)
USA	0 (0.0%)	127 (10.0%)	584 (46.7%)	1727 (39.0%)
Canada	0 (0.0%)	1143 (90.0%)	86 (6.9%)	213 (4.8%)
Previously treated with antimuscarinics	946 (49.6%)	714 (56.2%)	642 (51.3%)	2302 (52.0%)
Baseline mean number of micturitions per 24 h	11.6 (±2.9)	11.7 (±3.4)	11.6 (±3.1)	11.6 (±3.1)
Baseline mean number of incontinence episodes per 24 h	1.7 (±2.4)	2.1 (±2.7)	2.3 (±2.3)	1.8 (±2.5)

^a^BMI: Body mass index; USA: United States of America.

^b^SD: Standard deviation.

### Description of utilities according to symptoms severity

Mean utilities were 0.82 (±0.21) for EQ-5D and 0.86 (±0.09) for OAB-5D, for all patients and visits combined, with values ranging from-0.59 to 1 for EQ-5D, and from 0.61 to 1 for OAB-5D. A moderate correlation between OAB-5D and EQ-5D was found (r = 0.34, p < 0.0001).

Mean EQ-5D and OAB-5D utilities steadily decreased with increasing symptom severity. OAB-5D provided higher values than EQ-5D, whereas SD estimates were lower for OAB-5D (Table 2). Ranges of utilities from the most severe symptom level to the least were slightly larger for OAB-5D (0.10 for both micturitions and incontinence versus 0.07 and 0.09 for micturitions and incontinence levels, respectively for EQ-5D).

**Table 2:** Mean utility scores of the instruments and adjusted differences between the two utility scores according to symptom frequency level

**Clinical symptom**	**Symptom frequency level**	**Level definition**	**% per level**	**Mean utility (±SD)**^**a**^	**Mean utility (±SD)**^**a**^	**Difference between utilities, adjusted mean (±SD)**^**a**^	**P value**^**b**^
				**EQ-5D**	**OAB-5D**		
Micturitions/24 h	1	< 8	21.2	0.85 (±0.21)	0.90 (±0.08)	0.046 (±0.396)	<.0001
	2	8 - < =10	30.7	0.84 (±0.20)	0.87 (±0.09)	0.025 (±0.458)	<.0001
	3	10 - < =12	22.7	0.82 (±0.21)	0.85 (±0.09)	0.015 (±0.401)	0.0205
	4	12 - < = 14	13.2	0.80 (±0.22)	0.82 (±0.09)	0.007 (±0.325)	0.3073
	5	> 14	12.3	0.78 (±0.23)	0.80 (±0.09)	0.007 (±0.331)	0.3477
Incontinence episodes/24 h	1	0	50.3	0.85 (±0.19)	0.89 (±0.08)	0.032 (±0.470)	<.0001
	2	> 0 - < = 1	19.7	0.82 (±0.20)	0.85 (±0.09)	0.019 (±0.320)	0.0006
	3	1 - < =2	11.0	0.80 (±0.22)	0.83 (±0.09)	0.009 (±0.262)	0.1529
	4	2 - < =3	6.9	0.78 (±0.23)	0.81 (±0.09)	0.006 (±0.229)	0.4157
	5	> 3	12.2	0.76 (±0.26)	0.79 (±0.09)	0.004 (±0.290)	0.5353

^a^SD: Standard deviation.

^b^*t-*test to test the null hypothesis that the adjusted difference equals zero.

**Figure 1:**
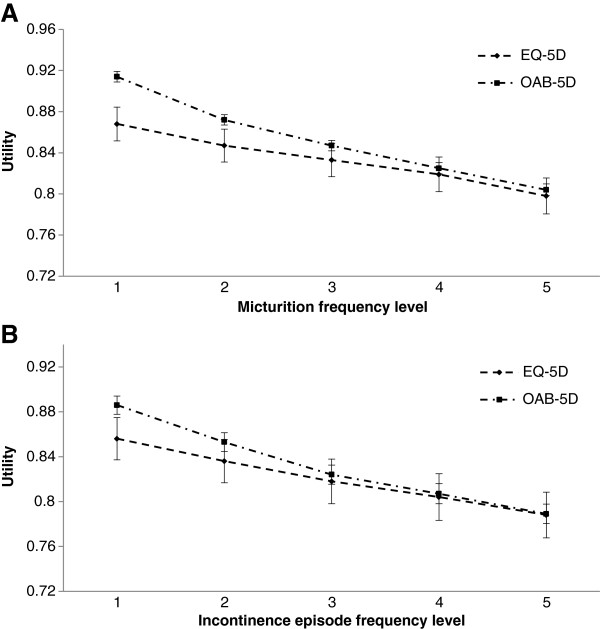
**Adjusted utility estimates of EQ-5D and OAB-5D according to symptom frequency level. A**–Adjusted utility estimates of EQ-5D and OAB-5D according to micturition frequency level; **B**–Adjusted utility estimates of EQ-5D and OAB-5D according to incontinence episode frequency level.

Adjusted estimates of utility by symptom frequency level are presented in Figure 1. For severe levels of micturition frequency (4 and 5), difference in utilities between the two instruments was not statistically significant. However, in the lower levels of severity (1 and 2), utilities generated by OAB-5D were higher and differences with EQ-5D were statistically significant (p < 0.0001) (Table 2).

Similar trends were observed for incontinence (Figure 1B). Utility values were not significantly different between EQ-5D and OAB-5D for the most severe levels of incontinence (3, 4 and 5). Differences in utilities between the two instruments became highly significant in less severe levels (1 and 2) (p-value < 0.001) (Table 2).

### Equations to predict EQ-5D and OAB-5D utilities from symptom levels

Age, gender, BMI, previous OAB treatment (yes/no) and study identifier were selected as adjustment variables in the regression models.

For EQ-5D utility modeling, the estimated RMSE for the linear model, the Beta model and the Tobit model were respectively 0.20829, 0.23819 and 0.26237. For OAB-5D utility modeling, the resulting RMSE for the linear model, the Beta model and the Tobit model were respectively 0.08000, 0.09245 and 0.09085. The linear model was consequently selected as the best model for both utility scores.

The estimated regression coefficients confirmed that utilities decreased with symptom severity after adjusting for patient characteristics (Table 3). Micturitions and incontinence appeared to have similar impact on EQ-5D, but micturitions had greater impact on OAB-5D utilities than incontinence. Improvement of incontinence level from 5 to 1 (i.e. from >3 incontinence episodes per day to 0) was associated with an increase of 0.052 in EQ-5D utility (p < 0.0001) and 0.076 in OAB-5D utility (p < 0.0001), at equal micturitions symptoms. Similarly, improvement of micturitions frequency from level 5 to 1 (i.e. from >7 episodes per day to to ≤1) was associated with an increase of 0.056 in EQ-5D utility (p < .0001) and 0.088 in OAB-5D utility (p < 0.0001), at equal incontinence symptoms.

**Table 3:** Equations to predict EQ-5D and OAB-5D utilities from symptom frequency level

**Effect**	**EQ-5D utility**			**OAB-5D utility**		
	**β estimate**	**β SE**^**a**^	**P value**^**b**^	**β estimate**	**β SE**^**a**^	**P value**^**b**^
Intercept	0.940	0.0230	<.0001	0.766	0.0085	<.0001
Age	-0.001	0.0002	0.0006	0.0003	0.0001	<.0001
Gender: female vs. male	-0.021	0.0063	0.0006	0.005	0.0024	0.0551
BMI	-0.003	0.0004	<.0001	-0.001	0.0002	0.0003
Previously treated with antimuscarinics	-0.019	0.0055	0.0006	-0.017	0.0021	<.0001
Micturitions level: 1 vs. 5	0.056	0.0057	<.0001	0.088	0.0022	<.0001
Micturitions level: 2 vs. 5	0.040	0.0052	<.0001	0.055	0.0021	<.0001
Micturitions level: 3 vs. 5	0.029	0.0051	<.0001	0.035	0.0020	<.0001
Micturitions level: 4 vs. 5	0.019	0.0051	0.0002	0.019	0.0020	<.0001
Incontinence episodes level: 1 vs. 5	0.052	0.0052	<.0001	0.076	0.0020	<.0001
Incontinence episodes level: 2 vs. 5	0.037	0.0052	<.0001	0.048	0.0020	<.0001
Incontinence episodes level: 3 vs. 5	0.022	0.0054	<.0001	0.025	0.0021	<.0001
Incontinence episodes level: 4 vs. 5	0.011	0.0058	0.0481	0.012	0.0023	<.0001
Study: SCORPIO vs. CAPRICORN	-0.019	0.0081	0.0174	-0.012	0.0030	<.0001
Study: ARIES vs. CAPRICORN	-0.008	0.0083	0.3409	0.004	0.0031	0.1984

^a^SE: Standard error.

^b^*t-*test to test the null hypothesis that the associated β estimate equals zero.

Utility values were estimated by the model for the least severe OAB state (level 1 for both symptoms) and the most severe (level 5 for both symptoms) OAB state and for average age (59.4 years), average BMI (28.9 kg/m2), gender (71.8% female), previous OAB treatment (52.0%) and study (43.1% SCORPIO, 28.7% ARIES, 28.3% CAPRICORN). These were respectively 0.870 and 0.762 based on EQ-5D (a difference of 0.108) and 0.923 and 0.759 based on OAB-5D (difference of 0.164).

The proportions of variance explained by the models (R^2^) were 26.1% for OAB-5D and 5.1% for EQ-5D.

### Responsiveness of HRQoL and clinical symptoms

More than half of the patients (56.4%) reported an improvement of at least 1 level in micturitions while 31.1% of them remained stable (all treatment arms combined). For incontinence episodes, patients who experienced improvement and those who remained stable represented similar proportions (46.3% and 45.6% respectively). Symptom worsening occurred in respectively 12.5% and 8.1% of patients for micturitions and incontinence episodes (Table 4A).

**Table 4:** Responsiveness of HRQoL and clinical symptoms

**A. Overall population**						
**Clinical symptom**	**Response in symptoms**	**N (%)**	**Change from baseline in utility EQ-5D, adjusted mean (±SD)**^**a**^	**P value vs. ‘Stable’**^**b**^	**Change from baseline in utility OAB-5D, adjusted mean (±SD)**^**a**^	**P value vs. ‘Stable’**^**b**^
Micturitions/24 h	Improvement ≥ 1 level	2286 (56.4%)	0.046 (±0.213)	0.0058	0.076 (±0.092)	<.0001
	Stable	1260 (31.1%)	0.027 (±0.202)	NA	0.039 (±0.087)	NA
	Worsening ≥ 1 level	506 (12.5%)	0.023 (±0.195)	0.7291	0.021 (±0.085)	0.0001
Incontinence episodes/24 h	Improvement ≥ 1 level	1874 (46.3%)	0.053 (±0.223)	0.0002	0.081 (±0.096)	<.0001
	Stable	1849 (45.6%)	0.028 (±0.206)	NA	0.042 (±0.089)	NA
	Worsening ≥ 1 level	329 (8.1%)	0.007 (±0.197)	0.0662	0.022 (±0.086)	<.0001
**B. Patients with mild symptoms at baseline (level 1 to 3 in terms of micturitions frequency, or incontinence episodes)**						
**Clinical symptom**	**Response in symptoms**	**N (%)**	**Change from baseline in utility EQ-5D, adjusted mean (±SD)**^**a**^	**P value vs. ‘Stable’**^**b**^	**Change from baseline in utility OAB-5D, adjusted mean (±SD)**^**a**^	**P value vs. ‘Stable’**^**b**^
Micturitions/24 h	Improvement ≥ 1 level	1329 (50.7%)	0.046 (±0.205)	0.3518	0.079 (±0.089)	<.0001
	Stable	863 (32.9%)	0.038 (±0.197)	NA	0.047 (±0.085)	NA
	Worsening ≥ 1 level	432 (16.5%)	0.029 (±0.189)	0.3886	0.021 (±0.082)	<.0001
Incontinence episodes/24 h	Improvement ≥ 1 level	1038 (35.9%)	0.049 (±0.197)	0.0416	0.079 (±0.099)	<.0001
	Stable	1564 (54.1%)	0.033 (±0.191)	NA	0.046 (±0.102)	NA
	Worsening ≥ 1 level	287 (9.9%)	0.010 (±0.188)	0.0628	0.023 (±0.087)	<.0001
**C. Patients with severe symptoms at baseline (level 4 or 5 in terms of micturitions frequency, or incontinence episodes)**						
**Clinical symptom**	**Response in symptoms**	**N (%)**	**Change from baseline in utility EQ-5D, adjusted mean (±SD)**^**a**^	**P value vs. ‘Stable’**^**b**^	**Change from baseline in utility OAB-5D, adjusted mean (±SD)**^**a**^	**P value vs. ‘Stable’**^**b**^
Micturitions/24 h	Improvement ≥ 1 level	957 (67.0%)	0.046 (±0.250)	0.0004	0.074 (±0.098)	<.0001
	Stable	397 (27.8%)	0.002 (±0.219)	NA	0.020 (±0.090)	NA
	Worsening ≥ 1 level	74 (5.2%)	-0.009 (±0.208)	0.6873	0.022 (±0.090)	0.8938
Incontinence episodes/24 h	Improvement ≥ 1 level	836 (71.9%)	0.064 (±0.299)	0.0004	0.078 (±0.143)	<.0001
	Stable	285 (24.5%)	0.012 (±0.246)	NA	0.014 (±0.111)	NA
	Worsening ≥ 1 level	42 (3.6%)	-0.019 (±0.219)	0.3865	0.008 (±0.094)	0.7158

A. ^a^SD: Standard deviation. ^b^*t-*test to test the null hypothesis that the associated utility change equals the utility change in patients with “Stable” response. NA: Not applicable.

B. ^a^SD: Standard deviation. ^b^*t-*test to test the null hypothesis that the associated utility change equals the utility change in patients with “Stable” response. NA: Not applicable.

C. ^a^SD: Standard deviation. ^b^*t-*test to test the null hypothesis that the associated utility change equals the utility change in patients with “Stable” response. NA: Not applicable.

In the overall population, mean changes from baseline in utility were positive in all groups of patients, those with improving, stable or worsening symptoms, and were greater in responders than in non-responders. There were no significant differences in change from baseline utility between worsening response and stable response for EQ-5D (p-values of 0.7291 and 0.0662 for micturitions and incontinence episodes respectively). However, differences became significant when using OAB-5D (p-value < 0.0001 for each symptom) (Table 4A).

In patients with mild micturition frequency (levels 1 to 3), the change from baseline in EQ-5D utility did not significantly differ between improvement and stable response (p-value of 0.3518) (Table 4B). When using OAB-5D, differences in utility were significant between improvement and stable response (p-value < 0.0001) as well as between worsening response and stable response) (p-value < 0.0001).

In severe cases, changes in micturition frequency and incontinence episodes were significantly associated with changes in EQ-5D utility. Thus, differences were significant between improvement and stable response (p = 0.0004 for both symptoms). Similar results were observed for OAB-5D (p < .0001 for both symptoms). No significant differences were observed between worsening response and stable response for both EQ-5D and OAB-5D (p-values of 0.3865 and 0.7158 for micturitions and incontinence episodes, respectively) (Table 4C).

Overall changes in utility from baseline values were greater with OAB-5D for all symptoms, in both improving and stable patients. In addition, differences in utility changes between improving and stable patients were higher with OAB-5D for each symptom (+0.037 with OAB-5D and +0.019 with EQ-5D for micturitions; +0.039 and +0.025 for incontinence) (Table 4A).

## Discussion

This study demonstrated that both utilities derived from EQ-5D and OAB-5D increased as OAB symptoms improved. Utilities were similar between the two instruments for severe levels of symptoms, but OAB-5D utilities were greater than EQ-5D utilities for lower levels of severity. Thus, the OAB-5D had a greater range of variation according to symptom severity. It is not surprising that, on average, EQ-5D utilities are lower than OAB-5D utilities since the generic instrument EQ-5D captures effects of patients’ co-morbidities on HRQL. The fact that incremental utilities between symptom levels are different between EQ-5D and OAB-5D is more noteworthy, considering that both instruments are expected to provide values on the same scale (where 1 represents perfect health and 0 a health state equivalent to death), and both sets of values are based on the time trade-off. The correlation between OAB-5D and EQ-5D in general population was moderate (r = 0.34; p < 0.0001). This is likely attributable to the noise around EQ-5D utilities, corroborated by the larger standard deviation around EQ-5D utilities, which may reflect natural variations in health status not related to OAB.

UK population norms for EQ-5D from a nationally representative interview survey of 3395 men and women aged 18 or over living in the UK were 0.81 (±0.26) for females and 0.78 (±0.28) for males aged from 55 to 64 years [20]. In the US, population norms for EQ-5D were 0.84 (SE of 0.01) for females and 0.86 (SE of 0.01) for males, in the same age group, 55 to 64 years [21]. In the studied population, the mean EQ-5D utility, based on UK TTO tariff, was 0.84 (±0.21). It is thus slightly higher than general population.

In a cost-effectiveness model evaluating tolterodine compared to no treatment in Sweden, Kobelt et al. [22] used a Markov model with five health states according to the number of micturitions and leakages per day, where state 1 was considered “mild” and state 5 “severe”. They obtained utility weights for the states by a linear regression analysis of the correlation between urinary symptoms and EQ-5D scores in a Swedish willingness-to-pay survey [7]. The following utility values were reported for different health states: level 1 (M + L <9): 0.742, level 2: (M + L < 12): 0.712, level 3 (12 ≤ M + L < 15): 0.676, level 4 (15 ≤ M + L < 18): 0.640 and level 5 (M + L ≥ 18): 0.598. Thus the difference between the least and more severe states was 0.144. Similarly, in this study, a model was provided to predict EQ-5D utilities for different health states, defined according to frequency of micturitions and incontinence episodes. EQ-5D utilities were higher, overall, ranging from 0.762 for the most severe state to 0.870 for the least severe states, but the difference in utilities of 0.108 between the two extreme OAB states was comparable to that reported by Kobelt et al.

In addition, an equation was provided to generate utilities for OAB states based on OAB-5D, with adjustment on age and gender. The two equations, based on EQ-5D and OAB-5D, can be used to derive utilities from clinical trials for which no utility instrument is provided. It should be noted that the predictive power of the EQ-5D equation is very low, whereas it appears acceptable for OAB-5D. This implies that a very large number of patients would be required to detect a difference in utility between different groups of patients using the EQ-5D equation. Another limitation of those regression models is that they do not capture potential effects of non-urological dimensions. Measuring the impact of adverse events on utilities using trial data was difficult since the periods during which patients experienced adverse events rarely coincided with dates of assessment visits. Furthermore, the OAB-5D system has no dimension related to the impact of adverse events of OAB treatments on HRQoL, whereas the EQ-5D, as a generic instrument, is expected to capture deteriotations in HRQoL related to adverse events.

An important finding based on the regression analyses performed in this study is that micturition frequency does affect the utility scores, even when adjusting on incontinence episodes. The interaction between micturition frequency and incontinence episodes was found not to be significant within the utility linear regression models (p = 0.1283), meaning that the numbers of micturition frequency and incontinence episodes have effects on utilities independently of each other. The effects of different symptoms were then assumed to be additive.

Both instruments were able to differentiate improving patients from stable patients. However the change in EQ-5D utility did not differ significantly between patients with improving or stable micturitions symptoms, among those with mild micturition frequency at baseline. Moreover, EQ-5D did not detect any significant difference between patients with stable symptoms and those with worsening symptoms in overall population. These findings highlight the greater responsiveness of OAB-5D as compared to EQ-5D, especially for patients with mild OAB. Additionnaly, this analysis revealed that utilities improved in stable patients and even in patients with worsening symptoms, among those with mild symptoms at baseline. Possible explanations for this unexpected trend would be that patients felt quite unwell at the time of entering the trial, and might have learned to better cope with their condition over time.

## Conclusions

In conclusion, this study showed that both EQ-5D and OAB-5D can detect changes in severity of OAB, especially in severe cases. Estimated differences in utilities between different OAB states are greater with OAB-5D, thus the choice of the instrument matters. Using EQ-5D to measure utilities has the advantage to generate values that can be compared to other conditions, and it can capture the impact of adverse events. However, large samples of patients are required to detect differences between different treatments or patient groups. EQ-5D may not be as sensitive as OAB-5D in measuring differences between treatments in milder cases of OAB. Contrary to EQ-5D, OAB-5D detects the difference between patients with worsening and stable symptoms. OAB-5D and EQ-5D can both be useful to derive utilities from clinical trial data and perform cost-effectiveness analyses in OAB, although they will lead to different incremental cost-effectiveness ratios.

## Competing interests

Astellas Pharma Europe Ltd provided financial support for this study.

## Authors’ contributions

KD, SA and KM designed the study. KD drafted the manuscript and carried out the statistical analyses. KD, SA, KM, JN, IO and ZH supervised the study and the interpretation of the results. All authors critically reviewed, contributed to and approved the final manuscript.

## Supplementary Material

Additional file 1 OAB-5D Classification System. Click here for file

## References

[B1] Abrams P Artibani W Cardozo L Dmochowski R Van Kerrebroeck P Sand P International Continence Society Reviewing the ICS 2002 terminology report: the ongoing debate Neurourol Urodyn 2009 28 4 287 10.1002/nau.20737 19350662

[B2] Brown JS McGhan WF Chokroverty S Comorbidities associated with overactive bladder Am J Manag Care 2000 6 Suppl 11 S574 S579 11183900

[B3] Irwin DE Irwin DE Milsom I Hunskaar S Reilly K Kopp Z Herschorn S Coyne K Kelleher C Hampel C Artibani W Abrams P Population-based survey of urinary incontinence, overactive bladder, and other lower urinary tract symptoms in five countries: results of the EPIC study Eur Urol 2006 50 6 1306 1314 discussion 1314-5 10.1016/j.eururo.2006.09.019 17049716

[B4] National Institute for Health and Clinical Excellence Guide to the methods of technology appraisal 2008 27905712

[B5] Gold MR Siegel JE Russell LB Weinstein MC Cost-effectiveness in health and medicine 1996 New York: Oxford University Press

[B6] Kobelt G Economic considerations and outcome measurement in urge incontinence Urology 1997 50 Suppl 6A 100 107 discussion 108-10 9426762 10.1016/s0090-4295(97)00602-x

[B7] Johannesson M O’Conor RM Kobelt-Nguyen G Mattiasson A Willingness to pay for reduced incontinence symptoms Br J Urol 1997 80 4 557 562 10.1046/j.1464-410X.1997.00420.x 9352692

[B8] Yang Y Brazier J Tsuchiya A Coyne K Estimating a preference-based single index from the overactive bladder questionnaire Value Health 2009 12 1 159 166 10.1111/j.1524-4733.2008.00413.x 18647258

[B9] Stolk EA Busschbach JJ Validity and feasibility of the use of condition-specific outcome measures in economic evaluation Qual Life Res 2003 12 4 363 371 10.1023/A:1023453405252 12797709

[B10] Eurich DT Johnson JA Reid KJ Spertus JA Assessing responsiveness of generic and specific health related quality of life measures in heart failure Health Qual Life Outcomes 2006 4 89 10.1186/1477-7525-4-89 17125512 PMC1675990

[B11] McKenna SP Ratcliffe J Meads DM Brazier JE Development and validation of a preference based measure derived from the Cambridge pulmonary hypertension outcome review (CAMPHOR) for use in cost utility analyses Health Qual Life Outcomes 2008 6 65 10.1186/1477-7525-6-65 18718016 PMC2546377

[B12] Young T Yang Y Brazier JE Tsuchiya A Coyne K The first stage of developing preference-based measures: constructing a health-state classification using Rasch analysis Qual Life Res 2009 18 2 253 265 10.1007/s11136-008-9428-0 19082759

[B13] Coyne K Revicki D Hunt T Corey R Stewart W Bentkover J Kurth H Abrams P Psychometric validation of an overactive bladder symptom and health-related quality of life questionnaire: the OAB-q Qual Life Res 2002 11 6 563 574 10.1023/A:1016370925601 12206577

[B14] Matza LS Thompson CL Krasnow J Brewster-Jordan J Zyczynski T Coyne KS Test-retest reliability of four questionnaires for patients with overactive bladder: the overactive bladder questionnaire (OAB-q), patient perception of bladder condition (PPBC), urgency questionnaire (UQ), and the primary OAB symptom questionnaire (POSQ) Neurourol Urodyn 2005 24 3 215 225 10.1002/nau.20110 15747340

[B15] Coyne KS Matza LS Thompson CL The responsiveness of the Overactive Bladder Questionnaire (OAB-q) Qual Life Res 2005 14 3 849 855 10.1007/s11136-004-0706-1 16022077

[B16] Khullar V Amarenco G Angulo JC Cambronero J Høye K Milsom I Radziszewski P Rechberger T Boerrigter P Drogendijk T Wooning M Chapple C Efficacy and tolerability of mirabegron, a β(3)-adrenoceptor agonist, in patients with overactive bladder: results from a randomised European-Australian phase 3 trial Eur Urol 2013 63 2 283 295 10.1016/j.eururo.2012.10.016 23182126

[B17] Nitti VW Auerbach S Martin N Calhoun A Lee M Herschorn S Results of a randomized phase III trial of mirabegron in patients with overactive bladder J Urol 2013 189 4 1388 1395 10.1016/j.juro.2012.10.017 23079373

[B18] Herschorn S Barkin J Castro-Diaz D Frankel JM Espuna-Pons M Gousse AE Stölzel M Martin N Gunther A Van Kerrebroeck P A phase III, randomized, double-blind, parallel-group, placebo-controlled, multicentre study to assess the efficacy and safety of the β(3)-adrenoceptor agonist, mirabegron, in patients with symptoms of overactive bladder Urology 2013 82 2 313 320 10.1016/j.urology.2013.02.077 23769122

[B19] Dolan P Jones-Lee M The time trade-off: a note on the effect of lifetime reallocation of consumption and discounting J Health Econ 1997 16 6 731 739 10.1016/S0167-6296(96)00514-0 10176781

[B20] Kind P Hardman G Macran S UK Population Norms for EQ-5D 1999 York Centre for Health Economics Discussion Paper 172. http://www.york.ac.uk/media/che/documents/papers/discussionpapers/CHE%20Discussion%20Paper%20172.pdf

[B21] Fryback DG Dunham NC Palta M Hanmer J Buechner J Cherepanov D Herrington SA Hays RD Kaplan RM Ganiats TG Feeny D Kind P US norms for six generic health-related quality-of-life indexes from the National Health Measurement study Med Care 2007 45 12 1162 1170 10.1097/MLR.0b013e31814848f1 18007166 PMC2647803

[B22] Kobelt G Jönsson L Mattiasson A Cost-effectiveness of new treatments for overactive bladder: the example of tolterodine, a new muscarinic agent: a Markov model Neurourol Urodyn 1998 17 6 599 611 10.1002/(SICI)1520-6777(1998)17:6<599::AID-NAU4>3.0.CO;2-J 9829424

